# Correlates of Cognitive SuperAging in the Mexican Health and Aging Study

**DOI:** 10.1002/alz.089747

**Published:** 2025-01-09

**Authors:** Joseph Saenz, Cassidy Doyle, Ross Andel

**Affiliations:** ^1^ Arizona State University, Phoenix, AZ USA; ^2^ University of South Florida, Tampa, FL USA; ^3^ Edson College of Nursing and Health Innovation, Arizona State University, Phoenix, AZ USA; ^4^ Memory Clinic, Department of Neurology, 2nd Faculty of Medicine, Charles University and Motol University Hospital, Prague Czech Republic

## Abstract

**Background:**

Aging is often accompanied by normative cognitive change, with memory decline typically experienced with advanced age. Some older adults reach their 80s maintaining memory performance of people 20 years younger. Little is known about these “SuperAgers” or correlates of SuperAging, particularly in low‐ and middle‐income settings. We evaluate how demographic, psychosocial, and health factors relate to odds of SuperAging in Mexico, an upper middle‐income country experiencing rapid population aging.

**Method:**

Data came from the Mexican Health and Aging Study, a nationally representative study of adults age 50+. We defined SuperAging as being 80+ years old, having delayed recall scores at or above the median for those age 50‐64, and not having cognitive impairment or dementia. We then explored associations between demographic, psychosocial, and health factors and odds of SuperAging.

**Result:**

Our sample included 334 individuals aged 80+ in the 2012 MHAS with no history of dementia or cognitive impairment, of whom 16% (n = 53) met criteria for SuperAging and 84% (n = 281) experienced normal cognitive aging. Women (OR: 1.90, p<0.05) were more likely to be among the SuperAgers, as were younger study participants (OR: 0.87, p<0.05), but years of education did not relate with SuperAging. Higher life satisfaction related positively with SuperAging (OR: 1.32, p<0.05) whereas other psychosocial factors (depressive symptoms and control beliefs) did not discriminate SuperAgers from their normal aging counterparts. Functional ability, chronic condition count, and self‐rated health were not related with SuperAging.

**Conclusion:**

Some individuals aged 80 and over maintain superior memory performance, with levels consistent with their peers in their fifties and early sixties. Using data from a study of older Mexican adults, we found that women and those reporting higher life satisfaction are more likely to be SuperAgers.

